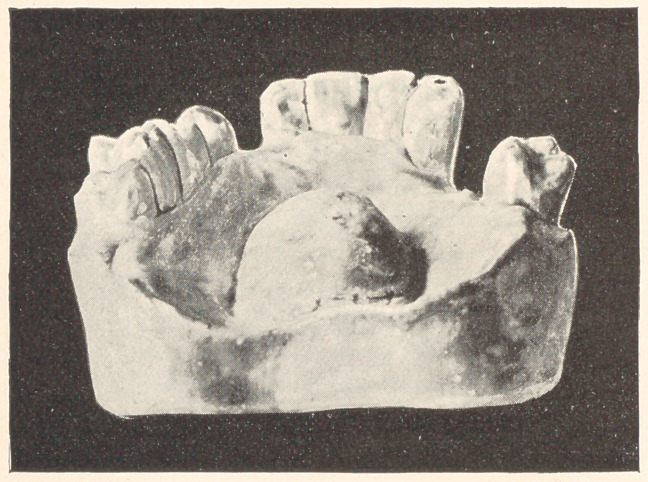# Some Observations on the Extraction of Teeth to Prevent Decay

**Published:** 1894-08

**Authors:** Eugene H. Smith

**Affiliations:** Boston, Mass.


### International Dental Journal.

Vol. XV. August, 1894. No. 8.

### Original Communications.[1]

### SOME OBSERVATIONS ON THE EXTRACTION OF TEETH TO PREVENT DECAY.[2]

BY EUGENE H. SMITH, D.M.D., BOSTON, MASS.[3]

Mr. President and Gentlemen,—The idealist has lived in all ages, playing an important part in the shaping of opinions. He exists at the present time and influences all conclusions. Rambler, to my mind, emphasized a great truth when he said, "There will always be a wide interval between practical and ideal excellence;" and it seems to me that one attains to the greatest good in this practical life of ours and in our professional work when he plans and puts into execution that which gives to his patients the greatest practical benefit.

Our literature abounds with varying opinions in regard to the extraction of the permanent teeth as a means to the prevention and correction of irregularities and the lessening of decay.

The models of the cases I show you to-night are not those where extracting was done for the correction of irregularities, but for the purpose of eliminating one of the great causes of early and exten-

---

[1] The editor and publishers are not responsible for the views of authors of papers published in this department, nor for any claim to novelty, or otherwise, that may be made by them. No papers will be received for this department that have appeared in any other journal published in the country.

[2] Read before the Harvard Odontological Society, March 29, 1894.

[3] Instructor in Orthodontia, Harvard University.

sive decay in teeth of poor structure,—that of lateral contact with pressure.

A gentleman, an idealist, writing on this subject thirty-five years ago, says, "There may be some extraordinary cases in which parting with a tooth does no harm," but generally the extracting of a bicuspid or a sixth-year molar destroys the whole character and beauty of the mouth, and that in a majority of instances the loss of these teeth is an unjustifiable interference with nature, and is virtually assuming that the all-wise Creator of the universe made a grand and radical mistake in the organization of the human family."

Could this writer to-day see the patients of our infirmaries, he could not help but conclude that a grand and radical mistake had been made, and that there really was something wrong somewhere, and only through the judicial extraction of some one or more teeth could improvement be made and the practical value of the teeth be maintained.

Opinions very similar are held and published to-day giving evidence of the idealist's presence. The other extreme, of extracting teeth in pairs and double pairs, is also held as the proper method of procedure; some going so far as to lay down exact rules as to the teeth to extract and the age at which they should be extracted.

Very great evil has been the result of improper extracting, and much mischief caused by the attempt to save too many teeth. It is from the observation of the widely different results that are formed such diversities of opinions. These opinions, however, lose very much of value when we consider that in a majority of the cases judged no models were made showing the condition before extracting, while models of the condition after extracting are presented in evidence of the mischief produced.

The few cases which I present to you this evening are from my private practice, where impressions of the mouth were taken and the conditions noted previous to extracting, and again after some lapse of time. The first case to which I ask your attention, represented by Models 24, A, B, and C, is that of a boy thirteen years of age; teeth of poor structure, very sensitive, and rapidly decaying. In October, 1889, the sixth-year molars were extracted, and they were chosen on account of their condition. You will observe that the teeth are not overcrowded and that the molars were extracted that the other teeth might drop back and thereby prevent decay of approximal surfaces. In this case, had the molars been in any good condition, I should have extracted four bicuspids. Model B shows the position of the teeth in March, 1890, and Model C

The models No. 24 will serve to illustrate the text, it not being deemed necessary to give all described in the paper.

No 24 - a

24 - C
24 - C

their position in 1893, with decay almost wholly stopped and the mouth in a thoroughly healthy condition.

The second case, Models 28, A, and B, is that of a young man, aged twenty-five years. Model A shows the condition in September, 1881, when I extracted the lower sixth-year molars. Some time during his teens he had had extracted the superior right sixth-year molar. You will observe how nicely the teeth are in line on that side, while on the other side, and on the lower, the teeth project and are much crowded. There was but little approximal decay on the side where the molar had been extracted, while all other approximal surfaces proved easy prey for its ravages.

Model B shows the position of the teeth when I extracted the upper left sixth-year molar, and which I feel now should have been done at the time I extracted the lower. Since then no impression has been taken, as the teeth are still moving back; slowly, of course, at his age. Approximal decay has diminished. In a short time I hope to take another impression showing the condition at the present.

Models 32, A, B, and C represent the case of a boy aged eleven years at the time of extracting the four sixth-year molars in November, 1889. They were selected on account of their very poor condition, as is shown in Model A. You will observe by these models that the teeth were affected by that malformation of their lower third due to the influence of some of the eruption diseases common to early childhood. These teeth were of poor structure and suffering from approximal decay. Models C and B show the change that has taken place,—the teeth slightly free from contact, and approximal decay absent. This case, as to the position of the teeth, is not quite all I could wish, on account of the upper incisors having dropped back to the extent of occluding directly on top of the lower incisors, but this condition is due to the fact that the malformed lower third of the upper incisors became broken off in an accident at foot-ball, preventing the over-shutting of the upper incisors to maintain the natural occlusion.

Models 34, A, and B. Case, a girl, aged thirteen years. Model A shows the condition at the time of the extraction of the three sixth-year molars in March, 1886, the other sixth-year molar having been extracted previously to the patient's coming under my care. The molars were selected in this case on account of their very poor condition, as their presence in the model testifies. The teeth of this young girl were of a very delicate structure and decaying rapidly.

Model B shows the change of position of the teeth in May,

1890. From the time of extracting, decay perceptibly diminished. You will observe that in the lower jaw the twelfth-year molar was erupted, while in the upper the twelfth-year molar had not appeared. In the upper the twelfth-year molar has come forward, maintaining an upright position, while in the lower the twelfth-year molar is slightly tipped forward, but not enough to mar a good and useful occlusion.

Models 67, A, and B show a case of a young miss fifteen years of age. The condition of the sixth-year molars is shown in Model A. They were extracted in April, 1890. It was at this time that the patient came under my care. Approximal decay was considerable, and the teeth were below the medium in structure. Had the sixth-year molar been in good condition, I probably should have extracted bicuspids, if any.

Model B shows the change that had taken place in October, 1893. Approximal impingement has been lessened just enough to change materially for the better the condition of decay. You will see by Model B how nicely the twelfth-year molars have come forward, without tipping, into the place of the sixth-year molars, giving the wisdom teeth an opportunity for usefulness rarely accorded them. The occlusion is well-nigh perfect, and a slight overlapping of the upper centrals has been righted.

Model 89. Case of a girl, aged fourteen years. Model A shows the condition in October, 1891, when the sixth-year molars were extracted. The teeth of this young girl are very delicate, and the molars were extracted to relieve the lateral contact and on account of their poor condition.

Model B shows the position at the present time,—decay very much lessened and the gum in a healthy condition. In all of these cases the sixth-year molars were the teeth extracted, for the reason that they were much poorer teeth than the others and in most of the cases decay had involved the pulp, and the probability of their being made useful teeth for many years was extremely doubtful. The present condition of these cases, as regards appearance, occlusion, and lessened liability to decay, is to my mind an endorsement of the extractions.

Much study should be given to all cases of this kind; and, in the absence of tabulated records, each case for the present must, in a measure, be a law unto itself.

If the teeth anterior to the sixth-year molars are very much crowded, so as to amount to an irregularity, I prefer to wait before extracting the sixth-year molars,—provided those are the teeth to be extracted,—until the twelfth-year molar is well in place, in order that the teeth may move back while the twelfth-year molar remains nearly in its eruption place. In case I wanted the teeth to move back but a little, I should extract the sixth-year molars just as the twelfth-year was appearing. This would allow the twelfth-year molar to move well forward into the place of the sixth-year, and at the same time allow the anterior teeth to separate sufficiently to lessen approximal decay.

In this paper I have not deemed it wise to enter into any elaborate discussion on the theory of extraction, but simply to present a contribution of recorded cases to a problem much discussed and still unsolved.

## Figures and Tables

**Figure f1:**
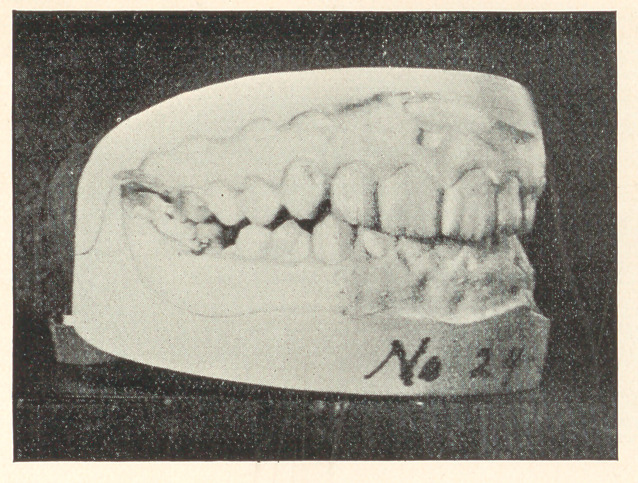


**Figure f2:**
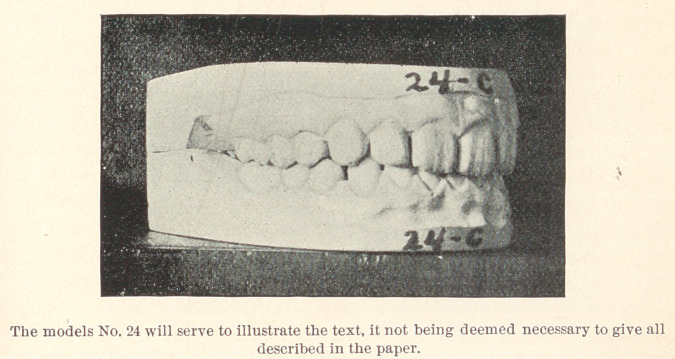


**Figure f3:**
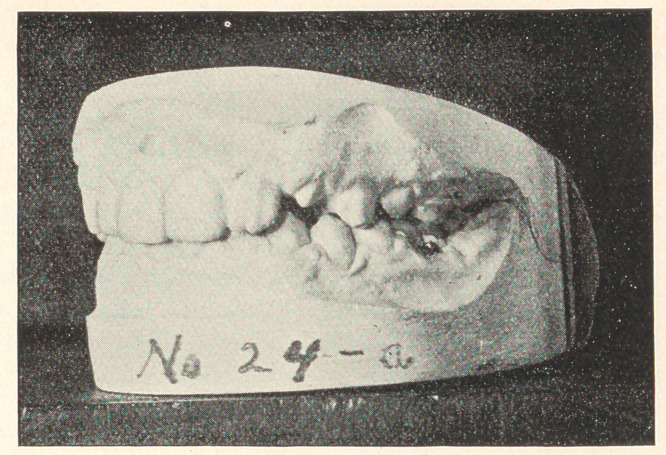


**Figure f4:**
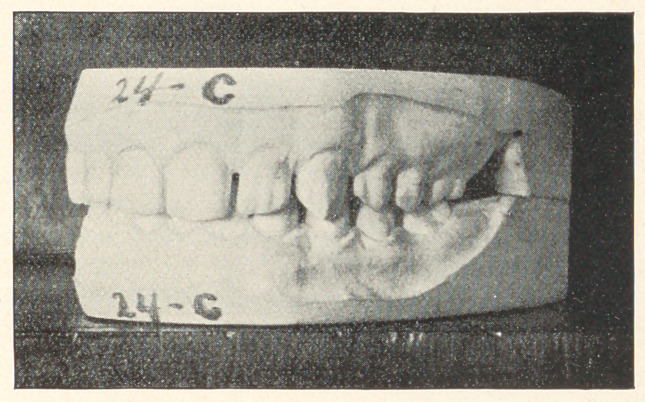


**Figure f5:**